# Monthly Summary

**Published:** 1891-07

**Authors:** 


### Monthly Summary.

PERMANENT ANTISEPTIC IRRIGATION.—Dr. E. von Meyer recommends this method of treating septic wounds, which he has successfully practised in Czerny's clinic during the last two years. It consists in placing the affected part in the metal tub, and keeping up constant irrigation by means of an irrigator provided with a spiral coil of perforated metallic tubing which acts as a "sprinkler."

The limb is lightly covered with gauze, and suspended in the tub by broad bandages attached to hooks at the sides. The bottom of the tub is sloping so that the fluid can flow off readily through an opening connecting with a tube, which terminates in a vessel placed under the bed. The cases in which this method has been employed comprise:

1. Cases of general sepsis arising from sub-fascial phlegmons and septic conditions after fractures.

(*a*) Cases of progressive gangrenous phlegmons, of spontaneous origin or developed after compound fractures.

(*b*) Septic amputation stumps after intermediate amputations and resections.

2. Extensive lacerated and contused wounds, with marked gangrene and sepsis of the soft parts.

On the ground of the results obtained in these cases, the author presents the following indications for the employment of permanent antiseptic irrigation:

1. In all cases of fractures and luxations attended with suppuration, especially when the surrounding parts are phlegmonous, and distinct signs of commencing sepsis are present.

2. In all cases of deeply seated progressive phlegmons in which gangrene is likely to occur.

3. In all cases mentioned above in which resection or amputation has to be performed, owing to pronounced general septic infection or for the treatment of septic amputation stumps.

In severe contused wounds in which a conservative method of treatment is selected, as soon as the integrity of the soft parts is threatened by gangrene, and the danger of a general septic intoxication is present.

For irrigation a number of antiseptic fluids have been proposed, but the best, in the author's opinion, is a 1 per cent. solution of aluminium acetate. In cases of septic and phlegmonous wounds the author also makes a liberal use of iodoform, to which he assigns an important part in the disinfection of aseptic wounds. The gauze layer should be changed twice every day, the wound carefully inspected, and all gangrenous fragments of tissue removed, so that the antiseptic solution comes in contact with a clean surface, washes away all pus, and penetrates the tissues. The drainage tubes in the wound must be carefully watched, for they become easily plugged with fragments of necrotic tissue. Unless all these details are attended to, no success can be expected from permanent irrigation; for the fluid then flows over firmly adherent necrotic portions, beneath which abundant pus stagnates. Permanent irrigation should only be discontinued when all signs of sepsis have completely disappeared, and the wound appears perfectly cleansed.—*Deut. Zeit. fur Chir.*

---

MANIPULATIVE TREATMENT AND DENTAL PATHOLOGY.—We pointed out in a recent article that Dr. Reyfuss had adopted with some success what he has called "the application of massage in the treatment of dental pathological conditions." We have already offered some explanation of the way in which this mode of treatment probably acts, and it only now remains for us to indicate how and in what cases it should be applied. Of course success must vary according to the case, and while massage may prove wholly satisfactory in some instances, it may upon the other hand only act as an adjuvant treatment in other cases, requiring to be supplemented by other methods. According to Dr. Reyfuss, it is useful in all local disturbances of the circulation, and impairment of nutrition. Inflammatory states, periostitis, gingivitis, alveolar abscess and neuralgias are also benefitted by massage of the gums, and this method of treatment may be employed, whether the pathological condition is incipient, in an acute or chronic state. As one would expect from what we said concerning its mode of action, massage is of especial value in local congestions, and when local irritation exists. We believe that in many cases massage should be assisted by the use of frictions with liniments or ointments, the basis of which should be lanolin as being more easily absorbed than other excipients. In cases in which much local tenderness exists, the use of a weak cocaine ointment employed in association with massage gives excellent results. Again, in some diseases of the periodontal membrane Dr. Reyfuss recommends massage, insisting that at first the treatment must be gentle, but when the formation of pus is imminent we are told to adopt more vigorous measures with a view to hastening suppuration and lessening the adjacent congestion. In the later stage when the abscess has emptied, the healing process is expedited by massage. In chronic abscess the tissues having taken on a languid condition, it is often extremely hard to excite a healthy action and to restore tone to the parts, and here our new remedy proves itself very useful, as it restores circulation and expresses the morbid accumulation of lymph which sodden the tissues. It is said also to prove beneficial in that troublesome class of cases when an abscess has formed and evacuated leaving a fistula which in its turn has closed, but pain and tenderness still persist. In cases of true neuralgia due to local irritation, climate changes, and generally when the nerve filaments are not too deeply situated massage will, we are assured by Dr. Reyfuss, exercise a most useful recuperative influence. Its *modus operandi* in these instances is said to be the same as when the nerve is "stretched," but whether this explanation will hold or not the relief obtained seems geuuine enough. Dr. Reyfuss, whose extremely interesting articles in the *Dental Mirror* will well repay reading, has used massage in over two hundred and fifty cases, and has met with most satisfactory results.—*Dental Record.*

MUSIC IN THE TREATMENT OF NERVOUS DISEASES.—Among the accessories of directly medicinal treatment, and side by side with such health-promoting agencies as rest, exercise, and change of surroundings, a definite place is due by right of its character and history to music. It is true that in later, as well as in earlier times, the sister art has been associated rather more with what is mythic and visionary in medicine than with its accredited or scientific practice. Miracle workers, mystics, and charlatans have in all ages made much of its influence on the minds of the impressible, whether truly suffering or not. There can be no doubt also that they have, as a class, been indebted in no small degree to their appreciation of this and the like natural aids for an apparent mastery of physical effects too commonly imputed to other personal and mysterious gifts of healing. In this respect they may teach us a lesson. The possession of a refined musical taste or a so called "ear" may be denied to many persons. Some conception of harmony is probably almost universal. This understanding differs, as it must, with different minds and moods, but it exists in some degree and form, we should say, in every human being. Each temperament knows and could show, if intelligence and language were adequate, the terms of its own simplest musical coefficient, and perhaps even some of its remembered variations. The cheerful rhythm that lightened for him an hour of gloom, the flowing cadence that absorbed his petulent irritation, are as the kindred spirits of a man's family. What wonder, then, if music be found equal to the treatment of some of his diseases—those, namely, which concern his mental and nervous condition. In connection with this subject Dr. G. H. Lilley sends us a short and interesting paper on the Therapeutics of Music in Mental Diseases. He discusses with considerable force the effect of this ancient remedy on those disorders of nutrition which explain so many departures from the normal health of mind and body. A brief and lucid argument illustrates the close resemblance in physiological action between music and other stimuli which regulate the vaso-motor activity employed in tissue nutrition. Some general observations on the mode of applying the treatment suggested are likewise worth reading, and the paper as a whole forms a useful and practical, if somewhat irregular, contribution to the hygiene of the nervous system.—*Lancet.*

---

TRIGEMINAL NEURALGIA AND IODIDE OF POTASSIUM.—In the last number of the *Neurologisches Centralblatt* reference is made to some interesting facts related by Dr. S. Ehrmann as to the occurrence of severe facial neuralgia after the administration of even small doses of iodide of potassium. In the first case mentioned the patient, a strong working man of thirty-five, suffered most intense pain in the forehead and in the teeth, with sensitiveness over the whole distribution of the fifth nerve, after taking fifteen grains of the drug. A second patient after taking thirty grains had much pain in the region of the upper jaw, with pain and tenderness in separate branches of the nerve, and also œdema of the eyelids on the left side. A third and a fourth patient also suffered from similar symptoms after similar doses. There were associated in all the cases with much lacrymation and injection of the conjunctiva, but the symptoms rapidly vanished, and did not reappear on a further administration of the drug. The cases are not only interesting, but important, for it is desirable to know as much as possible regarding any peculiar effects likely to be produced by a drug which is so frequently administered as an iodide of potassium.—*Lancet.*

